# Factors associated with dental students’ mental health during the COVID-19 pandemic: a longitudinal study

**DOI:** 10.1590/1807-3107bor-2024.vol38.0090

**Published:** 2024-09-13

**Authors:** Andréa Neiva da SILVA, Mario Vianna VETTORE

**Affiliations:** (a) Universidade Federal Fluminense – UFF, Institute of Collective Health, Department of Health and Society, Niterói, RJ, Brazil.; (b) University of Agder, Department of Health and Nursing Sciences, Kristiansand, Agder, Norway.

**Keywords:** Education, Dental, Psychosocial Functioning, Mental Health, COVID-19

## Abstract

The aim of the present study was to examine the relationships between gender, sense of coherence (SOC), social support, perception of academic environment and mental health of dental students during the coronavirus disease 2019 (COVID-19) pandemic. A longitudinal study involving 65 undergraduates enrolled from the first to sixth semesters in the bachelor dental course of Universidade Federal Fluminense, Brazil, was conducted. Baseline data (2018) included age, gender, SOC, social support, stress control and perception of education environment. Depression, anxiety and stress were assessed at baseline (response rate: 93%) and two-year follow-up (2020; response rate: 37%). Structural Equation Modelling was used to test the relationships between independent variables and depression, anxiety and stress during COVID-19 pandemic. Lower social support (β = -0.15), lower stress control (β = -0.20) and lower SOC (β = -0.39) were directly linked to higher depression (β = 0.22). Female gender (β = 0.22), worse perception of educational environment (β = -0.24) and lower SOC (β = -0.57) directly predicted higher anxiety. Female gender (β = 0.18), lower stress control (β = -0.21), and lower SOC (β = -0.46) directly predicted higher stress. The link between gender and both depression and stress, was mediated by stress control. Social support was indirectly linked to depression and stress via SOC. Perception of educational environment mediated the link between SOC and anxiety. Mental health of dental students during COVID-19 pandemic was influenced by demographic characteristics, perception of educational environment, social support and SOC through both direct and indirect pathways.

## Introduction

Coronavirus disease 2019 (COVID-19) pandemic emerged in Wuhan (China) in December 2019 and rapidly spread out across the globe due to the high transmissibility and infectivity of the SARS-CoV-2 virus. Restrictive social distancing measures were recommended to control the COVID-19 pandemic, including the interruption of university activities in several countries. During the first semester of 2020, 78% of public federal universities in Brazil suspended the academic activities, including the undergraduate courses.^1^ The pandemic affected undergraduate students’ mental health worldwide since they experienced increased levels of stress, anxiety, depression, and other mental health problems due to the various challenges posed by the pandemic.^2^ Peer social distancing, loneliness, fear, social media infodemic, sleep disorders, and the shift from face-to-face to online teaching impacted students’ psychological well-being.^3^

During the COVID-19 pandemic, dental students had higher levels of depression than undergraduate students from other courses.^4^ Difficulties in adapting to virtual learning, social isolation, and fear of infection have worsened the mental health of dental students during the pandemic.^5^ In the pre-pandemic period, studies have also demonstrated that high levels of anxiety and stress were common mental health problems among dental students.^6^ Depression manifests as a persistent feeling of sadness and decline of interest or pleasure in daily activities. Anxiety is characterized by a sense of anticipated fear of future life events.^7^ Stress is a process that involves the cognitive appraisal of external stimuli followed by individual behavioural and physiological responses.^8^

Individual and contextual characteristics, including self-efficacy, supportive academic environment,^9^ social support,^10^ and positive coping strategies, such as sense of coherence (SOC)^13^, can promote undergraduate students’ mental health. SOC is a global construct that reflects an individual’s overall perception and understanding of the surrounding environment, the ability to manage and cope with the stimuli they encounter throughout life and the capacity to give emotional meaning to these challenges.^11^ SOC was associated with lower levels of stress among university students.^12^ Social support refers to the individual perception and belief that one is esteemed, appreciated, and loved by their peers, relatives and members of their social network, which indicate the availability of social resources.^13^ Social support was correlated with SOC and psychological distress among undergraduate students.^10^ Academic environment can be defined as a learning setting encompassing access to learning resources, services, and facilities offered by educational institutions, that is also a place of development of social networks among students.^14^ Poor perception of the academic environment was associated with psychological distress amongst undergraduate students.^15^

A positive perception of the academic environment, high SOC, and social support may act as general resistance resources for dental students,^11^ which in turn may promote the development of coping strategies to deal with adversities, such as those imposed by the COVID-19 pandemic. Few longitudinal studies have assessed the role of these protective psychological factors and academic environment on dental students’ mental health during health emergencies. The investigation of individual and environmental resources that enhance psychological well-being can contribute to the development of strategies to promote the mental health of dental students during emergencies. This study aimed to investigate the relationships between gender, SOC, social support, academic environment and mental health of dental students during the COVID-19 pandemic.

## Methods

### Study design and eligibility criteria

A two-year follow up study with undergraduate dental students from the Dental School of the Universidade Federal Fluminense, Brazil, was conducted. All dental students aged 18 years or older from any gender, and regularly enrolled in the undergraduate dental course between the first and sixth academic semester in the Niterói Campus of the Universidade Federal Fluminense were invited.

### Study power calculation

The sample size of 65 participants would lend a power of 80% to detect effect size of at least 0.34 in a structural equation modelling analysis comprising two latent variables and seven observed variables, considering a significance level of 0.05.^16^

### Data collection and instruments

Baseline data was collected using self-completed questionnaires administered at the dental school’s classrooms, laboratories and dental clinics from August to October 2018. Initially, teachers, clinical and laboratory instructors from all academic semesters were informed about the aim of the study and data collection procedures as well as to schedule one day for data collection with their students. If necessary, at least three additional visits were conducted in order to reach the largest number of students who may have missed the first day of data collection.

The two-year follow up data collection was carried out in June 2020 during the COVID-19 pandemic. Baseline participants were invited to respond a structured online questionnaire using Google Forms. The academic semester at Universidade Federal Fluminense was interrupted in March 2020 as a result of social distancing measures due to COVID-19 pandemic. Thus, all face-to-face teaching activities have been suspended three months before the follow up data collection. The Students Union social media groups, including Instagram, Facebook and WhatsApp groups, were used to invite the dental students to participate in the follow up.

The baseline questionnaire consisted of sociodemographic characteristics, psychosocial factors and perception of education environment. Depression, anxiety and stress were assessed at baseline and two-year follow up. Sociodemographic characteristics included age, gender (male/female) and family monthly income (< 3 Brazilian minimal wages (BMW), 3-6 BMW, > 6-10 BMW and > 10 BMW).

Student’s perception of the academic environment was evaluated using the Brazilian version of the Dundee Ready Education Environment Measure (DREEM).^17,18^ The DREEM is a 50-item questionnaire answered on a five-point Likert scale comprising five subscales: ‘learning’, ‘teachers’, ‘academic’, ‘atmosphere’, and ‘social’. The DREEM scores vary from 0 to 200. The greater the DREEM score the better the perception of the teaching environment is.

Psychosocial factors were stress control, SOC and social support. The three items of the component ‘Stress control’ of the Individual Lifestyle Profile Questionnaire (ILPQ) were used to measure stress control.^19^ SOC was assessed with the Brazilian version of the SOC scale (SOC-13), consisting of 13 items followed by a five-point Likert scale.^20,21^ The sum of the SOC items vary between 13 and 65. Higher scores of SOC-13 indicate greater levels of SOC.^20^ Social support was assessed through the Medical Outcome Study (MOS) social support scale.^22,23^ The 19 items of the social support scale are grouped into five dimensions: material, affective, emotional, positive social interaction, and information. A higher score of social support scale suggests stronger social support.^22^

Depression, anxiety and stress were measured using the Brazilian version of the short form of the Depression, Anxiety and Stress Scale (DASS-21).^24,25^ The DASS-21 consists of 21 items assessed using a four-point Likert scale ranging from 0 = ‘strongly disagree’ to 3 = ‘totally agree’. Depression, anxiety and stress are independently assessed using seven items of the DASS-21each. The higher the score of DASS-21 the worse the psychological condition is.

Internal consistency of the questionnaires was assessed using Cronbach’s alpha coefficients. The results showed high reliability as follows: DREEM (α = 0.913), SOC (α = 0.747), social support scale (α = 0.943), depression DASS-21subscale (α = 0.831), anxiety DASS-21 subscale (α = 0.875), and stress DASS-21 subscale (α = 0.883).

### Data analysis

Descriptive analysis reported the distribution of the continuous and categorical variables through means (standard deviations) and proportions. Normal distribution of continuous variables was checked by Shapiro–Wilk test. The comparison of continuous and categorical variables between the analyzed sample and those lost in the follow up was assessed using Mann-Whitney test and Chi-square test, respectively. Related-samples Wilcoxon signed rank test was used to compare the scores of depression, anxiety and stress between baseline and during COVID-19 pandemic.

Confirmatory factorial analysis (CFA) was used to assess the multidimensionality of the latent variables student’s perceptions of the educational environment and social support. The scores of the five dimensions of DREEM questionnaire and the scores of the five dimensions of the social support scale were used as indicators for the latent variables student’s perception of the academic environment and social support, respectively.

**Figure 1:**
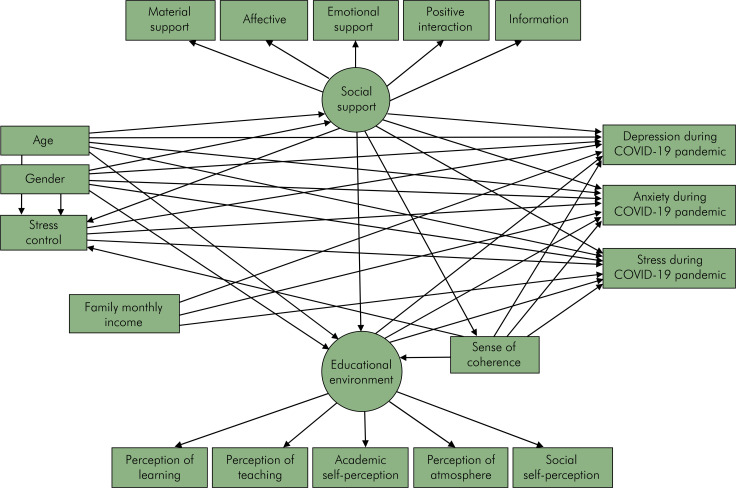
Theoretical framework of the associations between demographics, socioeconomic status, psychosocial factors and academic environment and dental students’ mental health.

The direct and indirect relationships between observed and latent variables were evaluated through Structural equation modelling (SEM) according to the proposed theoretical framework (Figure 1). Standardized direct effects and standardized indirect effects were estimated using AMOS SPSS, version 25.0. Maximum likelihood method via bias-corrected bootstrap was used to estimate the 95% confidence intervals (95% CIs) and to test mediation according to statistical significance of the indirect effects, with 900 resampling from the original data set to estimate less biased standard errors.^26^ Initially, the full model was estimated. Then, nonsignificant direct paths were removed, and the model was re-estimated to generate a statistically parsimonious model. The model fit of the measurement and structural models was assessed according to the following fit indexes and threshold values: χ2/df < 3.0, standardized root-mean-square residual (SRMR) ≤ 0.08, and comparative fit index (CFI) ≥ 0.90.^27^ Findings from statistical analysis were deemed significant if p < 0.05.

### Ethical aspects

The present study was in accordance with Helsinki Declaration and approved by the research ethics committee of the Faculty of Medicine of the Universidade Federal Fluminense, Niterói, RJ (Protocol no 4.132.396). All participants completed the self-administered questionnaire (baseline) and online questionnaire (two-year follow up) after providing informed consent.

## Results

The total number of dental students enrolled between the first and sixth academic semesters at the dental school at the Universidade Federal Fluminense in 2018 were 189. Of them, 175 accepted the invitation to respond the baseline questionnaire (response rate = 93%). Twelve students were excluded because they were aged less than 18 years. Two students were further excluded due to incomplete data. The two-year follow up data collection included 65 participants (follow up rate = 37%).

Sociodemographic characteristics, perception of education environment, stress control, SOC, and depression, anxiety and stress at baseline were not statistically different between the final analytic sample and participants lost during follow up. Social support total scores and material support scores were higher in the participants lost to follow up than those who completed the study period. The mean age of the analyzed sample was 21.1 years (SD = 2.1). Most dental students were females (84.6%). Around one-third of participants (n = 22) were from families with monthly income between 3 and 6 BMW (Table). Depression scores significantly increased between baseline and during COVID-19 pandemic (8.0 vs 9.6, p = 0.019), indicating a worsening in participants’ depression levels. Scores of anxiety (7.0 vs 8.1, p = 0.332) and stress (11.6 vs 12.1, p = 0.319) did not differ between baseline assessment and follow up.

**Table 1:** Comparison of participant’s sociodemographic characteristics, perception of education environment, psychosocial factors and depression, anxiety and stress at baseline, of the analytic sample participants and those lost to follow up.

Variables	Total sample at baseline (n= 175)	Participants who completed the study (n = 65)	Participants lost to follow up (n = 110)	p-value
Age, mean (SD)	21.5 (3.0)	21.1 (2.1)	21.8 (3.4)	0.154
Sex, n (%)				
Males	31 (17.7)	10 (15.4)	21 (19.1)	0.523
Females	144 (82.3)	55 (84.6)	89 (80.9)	
Monthly family income, n (%)				
< 3 BMW	46 (26.3)	18 (27.7)	28 (25.7)	0.532
3–6 BMW	60 (34.3)	22 (33.8)	38 (34.4)	
> 6–10 BMW	33 (18.8)	15 (23.1)	18 (16.8)	
> 10 BMW	36 (20.6)	10 (15.4)	26 (23.0)	
Educational environment (DREEM total score)	102.8 (23.5)	102.1 (22.5)	103.4 (24.1)	0.429
Learning	24.9 (6.4)	24.7 (6.8)	25.3 (6.5)	0.239
Teachers	23.6 (6.9)	23.6 (6.9)	23.5 (7.0)	0.989
Academic	16.0 (4.8)	16.0 (4.5)	15.9 (4.9)	0.827
Atmosphere	26.8 (6.5)	26.5 (6.1)	26.9 (6.6)	0.439
Social	11.6 (3.9)	11.4 (3.7)	11.8 (4.0)	0.531
Stress control	3.6 (2.2)	3.8 (1.8)	3.6 (2.3)	0.550
Sense of coherence	40.5 (7.3)	39.6 (6.5)	40.8 (7.7)	0.408
Social Support total score	80.9 (15.2)	78.1 (15.8)	82.3 (16.0)	0.049*
Material Support	15.2 (4.3)	14.3 (4.1)	15.8 (4.3)	0.011*
Affective	13.0 (2.6)	12.8 (2.6)	13.1 (2.7)	0.156
Emotional Support	15.4 (4.0)	14.9 (3.9)	15.5 (4.2)	0.248
Positive interaction	16.5 (3.2)	15.9 (3.5)	16.8 (3.2)	0.055
Information	15.5 (3.8)	15.1 (3.3)	15.7 (4.1)	0.099
Depression	7.0 (5.7)	8.0 (5.3)	6.6 (5.9)	0.078
Anxiety	6.1 (5.2)	7.0 (5.5)	5.7 (5.2)	0.132
Stress	10.9 (5.5)	11.6 (5.6)	10.7 (5.6)	0.290

p-value refers to Mann-Whitney test (continuous variables) and Chi-square test (categorical variables); *p < 0.05

**Figure 2:**
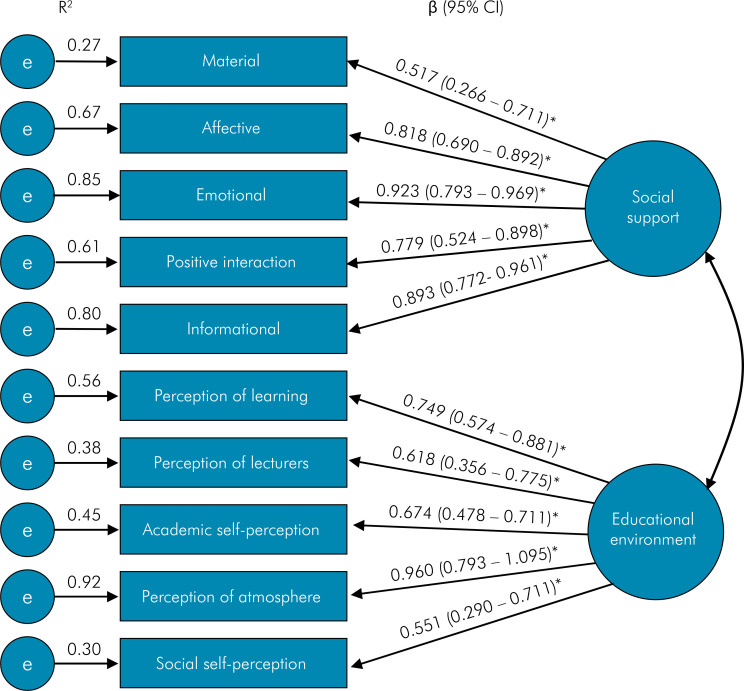
Confirmatory factor analysis of the two-factor and ten measurement model obtained through bootstrap item loadings (standard error/bias-corrected 95% CI). *p < 0.01.

The measurement, full and parsimonious models were acceptable fit to the data meeting the three of the a priori criteria. Measurement model: χ2/df = 1.521, SRMR = 0.075, CFI = 0.956; full model: χ2/df = 1.352, SRMR = 0.079, CFI = 0.932; parsimonious model: χ2/df = 1.375, SRMR = 0.077, CFI = 0.930. Confirmatory factor analysis (CFA) supported the perception of the educational environment and social support latent variables (Figure 2).

The full theoretical model was supported by the SEM. Monthly family income was not associated with any variable. Therefore, the variable monthly family income and non-significant direct paths were removed. The full model (M1) and parsimonious model (M2) did not differ (M2 vs. M1: ∆χ^2^(11) = 0.023, ns), suggesting the dropped variable and paths were not relevant to the model.

**Figure 3:**
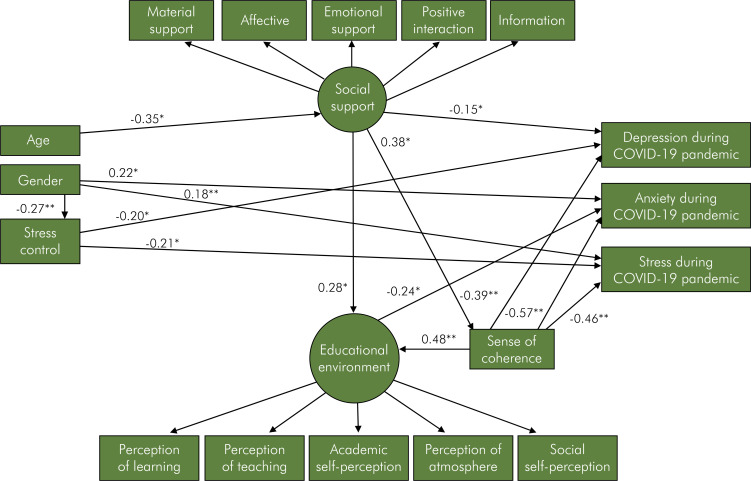
Direct effects for the parsimonious model. *p < 0.05, **p < 0.01

**Figure 4:**
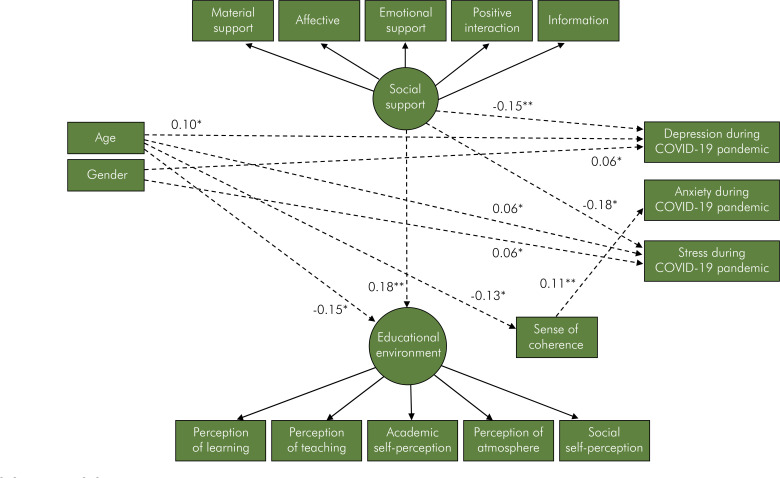
Indirect effects for the parsimonious model. *p < 0.05, **p < 0.01

The direct relationships between variables of the parsimonious model are described in Figure 3. Being older was linked to poor social support (β = -0.35). Female gender directly predicted less stress control (β = -0.27), higher anxiety during COVID-19 pandemic (β = 0.22) and higher stress during COVID-19 pandemic (β = 0.18). Greater stress control was linked to lower depression during COVID-19 pandemic (β = -0.20) and lower stress during COVID-19 pandemic (β = -0.21). Higher social support directly predicted better perception of the educational environment (β = 0.28), greater SOC (β = 0.38) and lower depression during COVID-19 pandemic (β = -0.15). Worse perception of the educational environment was directed linked to higher anxiety during COVID-19 pandemic (β = -0.24) Greater SOC was linked to better perception of the educational environment (β = 0.48), lower depression during COVID-19 pandemic (β = -0.39), lower anxiety during COVID-19 pandemic (β = -0.57), and lower stress during COVID-19 pandemic (β = -0.46). The indirect paths between variables are reported in Figure 4. Significant indirect associations of age, gender and social support with depression during COVID-19 pandemic and stress during COVID-19 pandemic were observed. Age and social support were indirectly linked to perception of the educational environment. Age was also indirectly linked to SOC.

Several indirect paths between non-adjacent variables were estimated by multiplying the standardised direct coefficients obtained in the parsimonious model. Age was linked to depression during COVID-19 pandemic via social support and SOC. Age was linked to stress during COVID-19 pandemic via social support and SOC. Stress control mediated the link between gender and depression during COVID-19 pandemic and between gender and stress during COVID-19 pandemic. SOC mediated the link between social support and depression during COVID-19 pandemic, and between social support and stress during COVID-19 pandemic. SOC was linked to anxiety during COVID-19 pandemic via perception of the educational environment.

## Discussion

The present study investigated the associations between demographic characteristics, SOC, social support, perception of the academic environment and mental health of dental students during the COVID-19 pandemic. A higher SOC was a relevant predictor of lower anxiety, lower stress and lower depression in the studied sample. SOC also mediated the link between social support and depression, and between social support and stress. Higher social support predicted lower depression. Moreover, female gender was directly associated with higher levels of anxiety and stress. Female gender also indirectly predicted greater levels of stress and depression via low control of stress. Younger dental students showed fewer symptoms of depression and stress via greater social support and higher SOC. In addition, those who had a better perception of the academic environment had lower anxiety during the COVID-19 pandemic.

The COVID-19 pandemic affected dental students’ academic routine and social life, decreasing their self-confidence and increasing fear and insecurity that consequently impacted their mental health. Given the threatening and uncertainties related to COVID-19 scenario, high SOC was associated with less psychological distress among undergraduate students from several countries.^28^ SOC may act as a resilient factor that protects students’ mental health. According to our findings, greater SOC also influenced better perception of the academic environment which, in turn, resulted in lower anxiety amongst dental students during the COVID-19 pandemic. A high SOC possibly allowed dental students to perceive a coherent, manageable and meaningful teaching environment, which promoted coping strategies to deal with stressors and, therefore, decreasing their anxiety levels.^28^

The direct effect of greater social support on lower levels of depression during the COVID-19 pandemic has already been reported among university students.^29^ In the present study, high social support predicted greater SOC among dental students. Moreover, SOC was associated with lower levels of stress, anxiety and depression. Support from family, friends and others facilitates the use of effective coping strategies to deal with the adversities during the pandemic^29^ and represents an important general resistance resource structuring SOC.^20^ A previous research also showed the mediating effect of SOC on adolescent’s mental health.^30^

Our findings showed that greater age and lower perceived social support were associated with depression. In addition, social support mediated the link between higher age and lower SOC. Low social support may have affected the capacity to cope effectively with stress during COVID-19, which may have contributed to greater psychological suffering.^31^ Previous studies have shown that younger individuals tend to present higher levels of psychological distress during the pandemic.^32,33^ In this study, older students possibly faced more difficulties in maintaining social ties via digital social networks, reducing their SOC and increasing their feeling of helplessness, and consequently increasing their levels of depression and stress. Social support is a general resource that structures SOC during youth.^20^Evidence suggests that the relationship between age and social support during the COVID-19 pandemic seems to be influenced by several factors.^34,35^

A range of biological, psychological, and gender-related factors explain the mental health differences between men and women. Biological discrepancies, including differences in hormones, functioning of the hypothalamic-pituitary-adrenal axis, and activity of the medial prefrontal cortex appear to contribute to the greater difficulty in controlling stress among women.^26,37^ Psychological differences between genders include cognitive styles, such as concerns about disapproval and the need for control, which may increase the risk of depression in women.^37^ Moreover, gender disparities in stress and anxiety were evident before the COVID-19 pandemic due to caregiving responsibilities, increased concerns about family and friend’s well-being, and the unmet need for social connectedness. Overall, these gender differences suggest that women are more affected by mental health problems than men.^33^ A recent review paper also indicated the link between female gender and higher psychological distress in dental students during the pandemic.^5^ Lower stress control mediated the relationship between female gender and higher levels of depression and stress in this study. A previous study suggested that women have experienced greater psychosocial overload than men during the pandemic in Brazil and other countries.^38^ The higher vulnerability to stress among female university students is probably related to the greater emotional sensitivity and concerns about the risk of SARs-COV2 infection than male students.^38^

A cross-sectional study carried out during the COVID-19 pandemic demonstrated the association between worse perception of online academic environment and depression among medical students.^39^ However, to the best of our knowledge no longitudinal study has investigated the influence of the perception of the academic environment in the COVID-19 pre-pandemic period on psychological distress during the pandemic. In the present study, greater social support was related to better perception of the academic environment in the pre-pandemic period. Moreover, the latter predicted lower anxiety among dental students during the COVID-19 pandemic. A similar finding was described in study conducted before the pandemic using cross-sectional data.^40^ A welcoming social atmosphere among medical students exerted a buffering effect on stress, favouring the perception of a more pleasant and enjoyable teaching environment, and thus benefiting students’ mental health.^39^

Educational strategies aiming at strengthening SOC, resilience, problem-solving and social skills throughout the course can benefit dental students’ mental health in pandemic situations. These strategies have also the potential to create a more welcoming academic environment resulting in a positive and lasting impact on their mental health, especially among those who are psychosocially vulnerable. Effective strategies to promote psychological well-being for undergraduate students during health emergencies periods through psychological support services should be made available very quickly. The above-mentioned approaches would be also relevant to enhance dental students’ mental health after post-pandemic periods since this particular group is highly susceptible to psychological distress throughout the course.^36^

The following limitations of this study should be acknowledged. Despite the high response rate at baseline, the follow-up data collection was composed of a small sample size. The uncertainties and worries about the COVID-19 pandemic as well as difficulties in reaching out participants at follow up through social media might explain the lower follow up rate. The sample included dental students enrolled in the first semesters of course in a public university in Brazil. Thus, our findings should be cautiously extrapolated to university students attending other courses as well the dental students in the final semesters. Moreover, potential changes in stress control, social support and SOC during the study period were not assessed. The main strength of the current study was the longitudinal design that allowed the evaluation of the predictors of mental health among dental students prospectively. Moreover, SEM is a robust statistical method employed to evaluate the direct and indirect relationships between variables simultaneously. Future research should address the complex relationships between psychosocial protective resources involved in coping with adversities at different stages of dental graduation after the COVID-19 pandemic.

## Conclusions

The increase of depression levels during the study period suggests that dental students’ mental health has worsened due to COVID-19 pandemic. Age, gender, perception of the educational environment, social support and SOC were meaningful factors that influenced the mental health of dental students during COVID-19 pandemic. Dental students would benefit from individual and collective interventions, including stress management strategies, counseling and psychological therapy to enhance their social life and mental health in order to mitigate the impact of COVID-19 pandemic. The aforementioned strategies should consider dental students’ gender and age group.
